# Perceived Benefits, Barriers, and Facilitators of a Digital Patient-Reported Outcomes Tool for Routine Diabetes Care: Protocol for a National, Multicenter, Mixed Methods Implementation Study

**DOI:** 10.2196/28391

**Published:** 2021-09-03

**Authors:** Søren Eik Skovlund, Antonio Nicolucci, Nina Balk-Møller, Dorthe B Berthelsen, Charlotte Glümer, Hans Perrild, Pernille Kjær, Lise Mellergaard Nørgaard, Lise Havbæk Troelsen, Anna Pietraszek, Danielle Hessler, Sherrie Kaplan, Niels Ejskjær

**Affiliations:** 1 Department of Clinical Medicine Aalborg University Aalborg Denmark; 2 Steno Diabetes Center North Jutland Aalborg University Hospital Aalborg Denmark; 3 Center for Outcomes Research and Clinical Epidemiology Pescara Italy; 4 PRO Secretariat, National Health Data Authority Copenhagen Denmark; 5 Department of Rehabilitation Municipality of Guldborgsund Nykoebing F Denmark; 6 Section for Biostatistics and Evidence-Based Research The Parker Institute Bispebjerg and Frederiksberg Hospital Copenhagen Denmark; 7 Center for Diabetes Copenhagen Municipality Copenhagen Denmark; 8 Department of Endocrinology Frederiksberg-Bisbebjerg Hospital Copenhagen Denmark; 9 Department of Family & Community Medicine University of California, San Francisco San Francisco, CA United States; 10 School of Medicine University of California, Irvine Irvine, CA United States

**Keywords:** diabetes, type 1 diabetes, type 2 diabetes, multisector care, digital health, patient-reported outcomes, patient-centered care, internet-administered, feasibility, mixed-method research, mobile phone

## Abstract

**Background:**

There is growing evidence that digital patient-reported outcome (PRO) questionnaires and PRO-based decision support tools may help improve the active engagement of people with diabetes in self-care, thereby improving the quality of care. However, many barriers still exist for the real-world effectiveness and implementation of such PRO tools in routine care. Furthermore, limited research has evaluated the acceptability, feasibility, and benefits of such tools across different health care settings.

**Objective:**

This study aims to evaluate the acceptability, feasibility, and perceived benefits of the Danish digital PRO diabetes tool in different health care settings in Denmark and to determine the factors affecting its implementation. Furthermore, the study evaluates the psychometric characteristics of the Danish PRO Diabetes Questionnaire and the validity of the scoring algorithms for dialogue support. The objective of this study is to guide the ongoing optimization of the PRO diabetes tool, its implementation, and the design of future randomized controlled effectiveness studies.

**Methods:**

We designed a multicenter, mixed methods, single-arm acceptability-feasibility implementation study protocol to contribute to the real-world pilot test of a new digital PRO diabetes tool in routine diabetes care. The use of the tool involves two main steps. First, the people with diabetes will complete a digital PRO Diabetes Questionnaire in the days before a routine diabetes visit. Second, the health care professional (HCP) will use a digital PRO tool to review the PRO results together with the people with diabetes during the visit. The PRO diabetes tool is designed to encourage and support people to take an active role for the people with diabetes in their own care and to expedite the delivery of person-centered, collaborative, and coordinated care.

**Results:**

A multicenter pilot study protocol and psychometrically designed digital data collection tools for evaluation were developed and deployed as part of a national evaluation of a new digital PRO diabetes intervention. A total of 598 people with diabetes and 34 HCPs completed the study protocol by April 1, 2021.

**Conclusions:**

A large-scale, mixed methods, multicenter study for evaluating the use of the nationally developed PRO Diabetes Questionnaire in routine care across all health care sectors in Denmark by using the RE-AIM (Reach, Efficacy, Adoption, Implementation and Maintenance) model as a framework has been designed and is ongoing. This study is expected to provide new important and detailed information about the real-world acceptability, perceived relevance, and benefits of the PRO diabetes tool among a large heterogeneous population of people with diabetes in Denmark and HCPs in different care settings. The results will be used to further improve the PRO tool, design implementation facilitation support strategies, and design future controlled effectiveness studies.

**International Registered Report Identifier (IRRID):**

DERR1-10.2196/28391

## Introduction

### Background

Patient-reported outcome (PRO) questionnaires and PRO-based digital decision support tools, henceforth referred to as PRO tools, may help improve the quality of life and multiple person-centered aspects of quality of care for those with diabetes when appropriately designed for use in routine practice [1-3]. Depending on the purpose, content, and design of the tool, digital self-assessment and PRO tools have the potential to increase person-centered care in many ways [4-7].

PRO tools may facilitate the active engagement of people with diabetes in caring for themselves on their own through improved self-insight and disease insight [8-10]; better preparation (of both the person with diabetes and health care professional [HCP]) before visits, thereby benefiting the quality of the visit [11]; focus on the person with diabetes’ individual needs and priorities [1]; detection of symptoms and underlying conditions requiring treatment [12-15]; assessment of symptom severity [16], prioritization of topics to discuss at the care visit [17]; monitoring of side effects [18,19] and treatment response; provision of treatment decision support [18]; and the creation of data allowing ongoing quality monitoring, benchmarking, and care improvement [20].

However, evidence shows that designing digital PRO tools that are acceptable, feasible, and effective among the majority of the population and successfully implementing them in diverse routine care settings are difficult tasks, owing to a variety of barriers to and challenges for both people with diabetes and HCPs [21-24]. The use of participatory research and the systematic involvement of patients in the design and evaluation of PRO tools has been emphasized as a means of improving the field’s knowledge and understanding the barriers to and facilitators for their sustainable use [1,2,25].

A national PRO diabetes tool comprising a PRO questionnaire and a digital clinical dialogue and decision support tool was developed in 2018-2020 [1] for use in routine diabetes care across health care sectors in Denmark as part of a national strategy to implement PRO in diabetes care. Its development was undertaken using a multi-stakeholder participatory and systematic stepwise approach involving both people with diabetes and HCPs across all stages to achieve an acceptable, person-centered, and feasible solution [1,26]. A detailed real-world evaluation of how people with diabetes experience the introduction of the new digital PRO diabetes tool in their routine care at a larger scale is important to guide the continued improvement of the tool. A multidimensional evaluation framework is required to evaluate the full range of potential factors influencing the reach, implementation, and effectiveness of the PRO tool [27,28].

This study is a multicenter PRO diabetes study (M-PRODIA) conducted in the context of a national evaluation of the newly developed national PRO diabetes tool under the auspices of the Danish Health Authority and the Region of North Denmark. The Danish PRO Diabetes Tool has two primary benefits. First, the tool is intended to support people with diabetes in becoming actively engaged in their own care and experiencing a greater influence on their care. Second, the tool was designed to improve the dialogue and quality of care visits by (1) improving the focus of care on what is most important to the individual person with diabetes; (2) enabling a structured and comprehensive review of people with diabetes’ biopsychosocial needs and priorities; and (3) facilitating a collaborative and coordinated approach to caring for people with diabetes.

Three broader strategic aims guiding the national value-based PRO diabetes program were to improve the delivery of coordinated, person-centered quality diabetes care for adults with type 1 and type 2 diabetes in Denmark, to allocate health services for optimal value to people with diabetes, and to collect PRO data to enable person-centered quality of diabetes care improvement and research.

The PRO diabetes tool consists of two elements: a newly developed diabetes questionnaire covering a wide range of topics relevant to people with diabetes (Multimedia Appendix 1) to be completed by people with diabetes before their routine visit [1], and a digital PRO tool that includes an interactive display of PRO results (PRO dashboard) for use by people with diabetes and HCPs together during the care visit.

In a 2019 study, we showed that in an outpatient clinic, the PRO diabetes tool was perceived as acceptable, feasible, and helpful in improving the active participation of people with diabetes and overall quality of the dialogue [29]. Further evidence involving a broader group of people with diabetes and HCPs is required to evaluate the validity, reliability, acceptability, feasibility, and effectiveness of the PRO diabetes tool and identify knowledge gaps, guide future research, and inform the planning of wider implementation. Research on the acceptance of digital PRO solutions among people with diabetes highlights that multiple possible issues may affect acceptance, including eHealth literacy, privacy concerns, emotional impact [24], and factors such as expected personal benefit, expected ease of use, perceived social acceptance, and facilitating conditions (user-centered design for use) [30]. It is hypothesized that this PRO diabetes tool, due to the use of systematic patient involvement [31,32] in the design phase to address these factors, will have a high real-world acceptance among people with diabetes. Few studies similarly highlight a range of possible barriers to the optimal adoption and use of PRO tools in diabetes care by HCP [33-35]. As a national multidisciplinary group of HCPs was involved in all stages of the development of the PRO diabetes tool with attention to these issues, it is hypothesized that there will be a high level of adoption of this tool among people with diabetes. Defining the prerequisites for effective use by HCPs, such as indicators for fidelity, skills training, and support, will be significant in guiding the implementation of the tool [33].

Furthermore, it is necessary to confirm the extent to which the PRO questionnaire meets overall quality criteria for clinical PRO tools [1], including (1) its acceptability for both people with diabetes and HCPs, (2) its usefulness and relevance across the care continuum, (3) its support for active engagement of the people with diabetes, and (4) its contribution to person-centered care outcomes (eg, care experience, care quality, care satisfaction, health-related empowerment, and health and diabetes-related quality of life outcomes) [36].

It is essential to evaluate the extent to which the PRO tool influences process indicators for person-centered diabetes care, such as the quality of communication and interpersonal relationships, collaboration, and the use of shared decision-making. The introduction of PRO may affect the quality of person-centered diabetes language and communication among people with diabetes, people with diabetes and family members of people with diabetes, among HCP within and across teams, and among health care sectors [37-39]. Furthermore, PRO may affect the quality of preventive and health-promoting activities, the quality of medical diabetes care and self-management support [1], and the consideration of the voice of people with diabetes in payer decisions on health care. It is relevant to evaluate the extent to which the PRO tool impacts individual factors such as the health competency, health-related empowerment, active engagement, and self-management behaviors of the people with diabetes; the experiences of people with diabetes with regard to care and support; and patient-relevant treatment outcomes.

Detailed empirical data, both qualitative and quantitative, are needed regarding the process of implementation in practice, identifying the key barriers to and facilitators for its effective implementation, its reach among the adult diabetes population, and the intervention requirements to fulfill the tool’s purpose and exert its intended effect at the population level [40]. To our knowledge, the Danish PRO Diabetes Tool is the first digital multidimensional PRO Diabetes Questionnaire, specifically designed through a systematic multi-stakeholder participatory process to improve the quality of person-centered care across primary, secondary, and municipality care settings. This tool was designed from 2017 to 2020 through a stepwise national participatory process including 5 multi-stakeholder, multidisciplinary full-day meetings with the representation of people with diabetes, payers, patient groups, health care sectors, and geographical regions in Denmark and 7 workshops with people with diabetes and ongoing clinically anchored partnering with people with diabetes.

Therefore, there is a need to evaluate the multi-faceted potential of the PRO diabetes tool to improve care quality and benefit people with diabetes through multiple pathways. This includes but is not limited to the active involvement of people with diabetes in their care, the facilitation of health literacy and health-related empowerment [41], early detection and preventive care [42,43], dialogue and decision support, outcome monitoring, value-based person-centered care, communication, culture, and organization for patient-centricity and chronic illness care coordination.

Examining the usability, acceptability, benefits, psychometric reliability, and validity (face validity, content validity, construct validity, and discriminatory validity), sensitivity, and responsiveness of the tool in different care settings and patient subgroups is required to ensure quality and optimize scaling and planning for implementation. This involves disentangling the complex interdependencies influencing reach, adoption, efficacy, and institutionalization through the use of mixed methods research [2].

The RE-AIM (Reach, Efficacy, Adoption, Implementation and Maintenance) model has been found to be helpful in previous diabetes research to evaluate person-centered diabetes initiatives and digital health interventions [40,44,45]. We adapted this model to facilitate the integration of many factors that influence the public health potential of an intervention.

The M-PRODIA is designed to piggyback on a pilot test program in routine care, as it is not possible to establish attention-control groups. Instead, considering feasibility, at one site, substudies were designed in parallel to compare PRO visits with regular visits in relation to follow-up care and health care use parameters and examine longitudinal changes in clinical care and outcomes. These have been reported in separate protocols. The M-PRODIA aims to use both quantitative and qualitative methods to characterize the real-world experiences of using the national Danish digital PRO diabetes tool among a large heterogeneous population of people with diabetes in Denmark and HCPs in different care settings.

### Study Objectives

The primary objective of this study is to evaluate the feasibility and acceptability of the PRO diabetes tool in practice and to explore and characterize its perceived benefits, risks, and disadvantages in routine diabetes care as part of a national pilot study. The study is designed for formative research purposes [46], using both exploratory and confirmatory approaches to inform the design of future research, such as potentially stepped-wedge [47] and randomized controlled protocols for the examination of public health and cost- and clinical effectiveness [46]. Furthermore, it aims to explore the individual, HCP, and system-level factors that may significantly influence different aspects of reach, implementation, and effectiveness.

The M-PRODIA’s specific research objectives are fivefold, as follows:

1. The study aims to assess and compare the perceived benefits and disadvantages related to the use of the PRO diabetes tool for dialogue and decision support in different care settings in a diverse population of people with diabetes.
2. The study aims to identify barriers to and facilitators for the optimal use of the tool from both the viewpoints of people with diabetes and HCPs.
3. The study aims to evaluate the validity, reliability, and clinical utility of the tool PRO Diabetes Questionnaire and Tool.
4. The study aims to obtain data to check for errors and optimize the PRO Diabetes Questionnaire and digital support tools.
5. The study aims to obtain the initial experience with estimation of RE-AIM indicators and guide future RE-AIM evaluation studies of the PRO diabetes tool.

## Methods

### Study Design

The M-PRODIA is a pragmatic, single-arm, real-world, mixed methods formative feasibility-acceptability pilot implementation study.

### Involvement of People With Diabetes in the Research Design

A panel of 3 persons with type 2 diabetes and 2 persons with type 1 diabetes all with experience as advocates for the perspective of people with diabetes were involved as collaborators in the design of this study and its study materials through regular working meetings with the research team. Several participants were involved in ongoing patient association activities and contributed with personal as well as collective insights regarding the perspective of people with diabetes on study questions. Meetings continue to be held regularly to ensure input to all phases of the study including study questions, study materials and questionnaires, and interpretation and dissemination of study results in line with seven quality criteria for patient involvement [26].

People with diabetes and family members of people with diabetes were systematically involved in all stages of the collaborative development and design of the national PRO Diabetes Questionnaire and the digital PRO diabetes tool, DiaProfil [1]. A total of 7 quality criteria for guiding involvement of people with diabetes were agreed upon between the Value Based Health Care and PRO in Diabetes Project (VBHC-PRO-DIA) research team and people with diabetes from the beginning [26]. The involvement of people with diabetes in the design of the PRO tool was undertaken as part of a separate embedded research study to develop and evaluate methods for patient involvement in clinical PRO tools design. All people with diabetes partnering on the development of the PRO diabetes tool completed informed consent for this research study. Results regarding the outcomes and impacts of involvement of people with diabetes will be analyzed and disseminated separately [48].

### Study Setting

The PRO diabetes tool was tested in three different health care settings, as shown in Table 1.

**Table 1:** Characteristics of study settings.

Settings	Target group characteristics	Primary use of PRO^a^
Hospital outpatient diabetes clinics	Type 1 diabetes Type 2 diabetes referred due to complexity and treatment burden	PRO data are mainly used in annual diabetes visits (30-60 minutes) with a specialized the diabetes nurse. Physicians may also use it during initial medical visits with people with diabetes (40 minutes).
Municipality rehabilitation service centers	Type 2 diabetes referred from general practice for lifestyle, health promotion, and diabetes education	The PRO is used during initial start-up visits at municipality centers and is potentially used during 3-month follow-up evaluation visits by multidisciplinary HCPs^b^ (dietitians and physiotherapists).
Primary care	Mainly type 2 diabetes treated regularly in primary practice	The PRO tool is used in regular routine and on-demand visits in primary practice settings (physician and nurse).

^a^PRO: patient-reported outcome.

^b^HCP: health care professional.

### Eligibility Criteria

Participants were eligible for this study if they were (1) adults with type 1, 2, or other type of diabetes; (2) able to read and understand Danish; and (3) scheduled for a diabetes visit during the study period. Participants were excluded if they had another severe illness that would make their participation impossible. In one hospital, people with diabetes would also be excluded if they had been diagnosed with diabetes less than 12 months ago.

### Description of the PRO Diabetes Intervention

#### Overview

The PRO Diabetes Questionnaire and its scoring algorithms for clinical use were developed through a national participatory design process to create a psychometrically valid tool that would be feasible for use in routine care to increase the delivery of person-centered diabetes care [1,26,49].

The questionnaire design was guided by participatory cocreation processes with people with diabetes and HCPs, using qualitative research [1] and extensive literature review to take into account empirical research related to diabetes self-efficacy [50], self-determination [51], empowerment [52], social ecological and biopsychosocial care [53], behavioral and health psychological diabetes research [43], and person-centered diabetes care [1].

**Figure 1:**
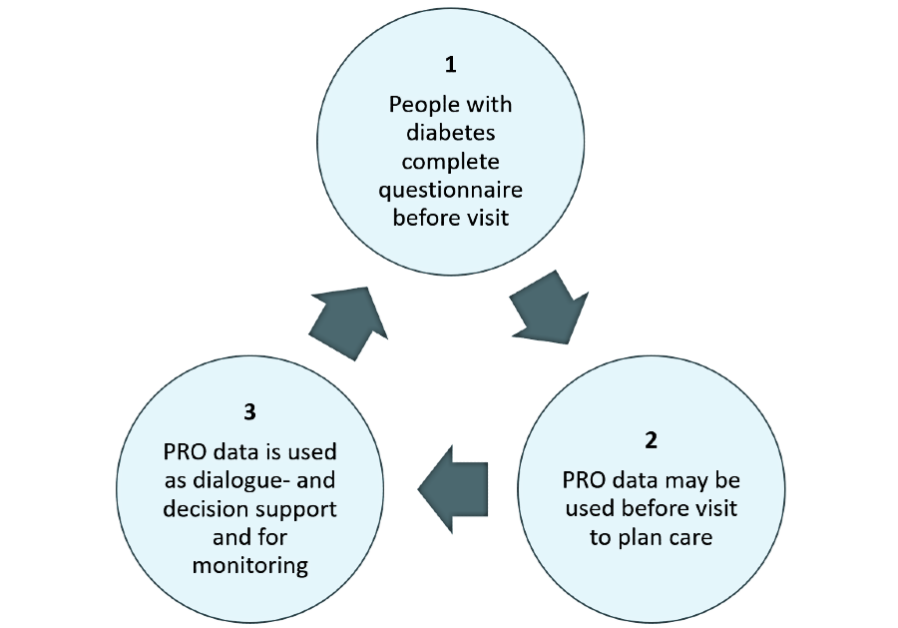
Key components of the PRO diabetes intervention used in all care settings. PRO: patient-reported outcome.

The core elements of the PRO diabetes intervention used across care settings are shown in Figure 1.

Initially, people with diabetes completed the PRO Diabetes Questionnaire at home using their smartphone, tablet, or computer preferably 2-10 days before their scheduled diabetes visit at a clinical care center or municipality diabetes center. This is intended to facilitate an optimal dialogue with an enhanced focus on the most important priorities of people with diabetes. The questionnaire measures generic and diabetes-specific topics that can only be reported directly by people with diabetes. The topics were established as important and relevant for both people with diabetes and HCPs. The HCP actively uses the PRO results during the care visit, together with the person with diabetes. A digital clinical PRO tool is used, which includes a dashboard that shows all the PRO results, to facilitate a review of issues and setting of priorities as part of a collaborative dialogue. The HCP had the technical option to access the PRO results of the people with diabetes through the HCP interface of the digital PRO tool once people with diabetes completed the questionnaire. However, upon completion of the questionnaire, it was explained to the person with diabetes that the results will be reviewed by the HCP just before the visit. If the person with diabetes needed support before the visit, they were advised to contact the HCP by phone or email.

Each PRO question answered by the people with diabetes is scored using a predefined scoring algorithm so that the results can be indicated in the digital PRO tool by green, yellow, or red colors. The scoring algorithms were defined through an iterative process involving HCPs, people with diabetes, and researchers to optimize clinical utility and validity. A green score indicates that there may be no problem for the responder on that item, a yellow score indicates the presence of concerns or issues requiring attention, and a red score indicates that there is a likely need for action and that the HCP and person with diabetes should address the topic. The digital PRO tool also provides easy access to raw data or scale scores if preferred by the people with diabetes or HCPs.

Before the study, each participating study site identified the most suitable way to fit the PRO tool into their existing care visits, which resulted in minor differences in the application of the tool while preserving the aim of delivering a common intervention.

All study sites participated in collaborative meetings to exchange experiences and approaches across the sites. An outline of the generally agreed approach to recommended person-centered use of the PRO Diabetes results is shown in Multimedia Appendix 2.

Two different digital software systems were used to provide the functionality defined by the PRO Diabetes Questionnaire and its scoring algorithms, the DiaProfil PRO diabetes tool developed by the VBHC-PRO-DIA at Aalborg University Hospital, and the health platform, EpicCare, the working tool of the hospitals in two of five Danish regions.

#### The Danish PRO Diabetes Questionnaire

The Danish PRO Diabetes Questionnaire includes 33-71 items and covers a range of carefully selected and defined health-related constructs.

The key content categories of the PRO Diabetes Questionnaire are shown in Textbox 1.

**Overview of the main content categories of the Danish Patient-Reported Outcomes Diabetes Questionnaire (2020). Constructs, short descriptions, and examples of content are presented.**

**General health and life situation**

- Self-assessed general health, social support, and life stressors affecting diabetes management

**Mental well-being**

- Positive psychological well-being and depression symptoms

**Symptom distress**

- Distress related to pain, heart, gastrointestinal, sexual dysfunction, and sleep and foot problems or symptoms

**Daily life with diabetes**

- Fitting diabetes into daily life and diabetes-specific social support

**Worries due to diabetes**

- Worry about disease progression, that is, diabetes complications

**Diabetes self-management confidence**

- Confidence in managing diabetes (diet, physical activity, adjusting treatment, self-monitoring, and care seeking)

**Blood sugar regulation**

- Perceived quality of blood sugar regulation and burden due to hypoglycemia and the fluctuation of blood sugar levels

**Medicine experience**

- Efficacy, convenience, side effect distress, and satisfaction

**Access to care**

- Confidence in ability to get in contact with a health care professional if needed in relation to diabetes

**Personal priorities for diabetes care**

- Wish for support for specific aspects of self-management
- Priority topics to discuss at the diabetes visit

The questionnaire consists of a combination of previously psychometrically validated items and scales, as well as newly adapted or designed items. Adaptation or design of new items was only done if no previously validated items were available that fit the requirements of people with diabetes and HCPs. In addition, a participatory and qualitative approach was used, including literature review, desk research, and qualitative research for each construct. It uses branch logic, so each person with diabetes only receives directly relevant questions. The psychometric validity, reliability of each item, and appropriateness of the scoring algorithms and logic rules will be examined in this study.

#### The Digital PRO Tool: DiaProfil

DiaProfil is a stand-alone PRO diabetes digital tool, codeveloped with the inputs of people with diabetes and HCPs by Aalborg University Hospital with the support of Zitelab, specifically for augmented use of the national PRO Diabetes Questionnaire as a tool for person-centered care delivery across care settings. An iterative, user-centered design process was undertaken involving people with diabetes and a multidisciplinary HCP team at Aalborg University Hospital to define optimal functionality and user interfaces. Previous experiences with other multidimensional PRO tools for diabetes were also considered [54].

DiaProfil allows the person with diabetes to complete the PRO questionnaire using mobile, tablet, or a PC and provides the HCP with web-based access to a multi-layered interactive PRO dashboard and an administrative system for reviewing and ordering PRO assessments. A significant new functionality in DiaProfil, resulting from the participatory design process, was the integration of actionable information for each PRO construct into the dashboard. The tool provided the HCP with information about available treatment and referral options, community services, and educational materials for each PRO construct. It required the clinical team to undertake an extensive desk-research exercise to map all resources and follow-up actions for each construct beyond its own care setting.

The DiaProfil dashboard was designed to provide an intuitive overview of the people with diabetes’ overall PRO results in one screen to allow HCPs to achieve an overview almost instantly, which can be useful in daily practice. Furthermore, the tool was developed for acceptability and readability for people with diabetes to facilitate collaborative use by people with diabetes and HCPs together on equal terms during the visit.

A screenshot of the DiaProfil PRO dashboard is shown in Multimedia Appendix 3.

The dashboard presenting the results is interactive and contains multiple layers of information that are accessible with one or two mouse clicks from the main screen. For each PRO topic and output, key information is obtained with a single click, including (1) dialogue tips and tools, (2) educational materials, (3) referral options, and (4) listing of locally relevant care and support options. For instance, if a person with diabetes likely had depression, the HCP could click on the topic on the screen to directly view information about local referral options and various psychosocial support and self-help resources.

DiaProfil was used in all participating study sites, except for one hospital that used the health platform tool.

#### The Danish Health Care Platform: Sundhedsplatformen

To integrate PRO data into their existing health information technology infrastructure, one hospital, Frederiksberg-Bispebjerg, used their existing generic IT health platform, EPIC, to collect and display PRO data. This platform is used by all hospitals covering 2.6 million inhabitants. In this hospital, people with diabetes completed the PRO Diabetes Questionnaire using the existing *My Health* app, which patients in the Capital Region of Denmark use to access general health care information. In this system, the HCP can view PRO results on multiple data screens that combine clinical and PRO data. Detailed methods for displaying PRO data on the clinical screen and ensuring proper placement in the patient flow were developed by the clinical diabetes care team together with the health information technology provider (Center for IT, Medico and Telephony) for optimal usability and integration into the existing workflow.

### Outcomes

#### Working Model for the Evaluation of the Process and Outcome Indicators

**Figure 2:**
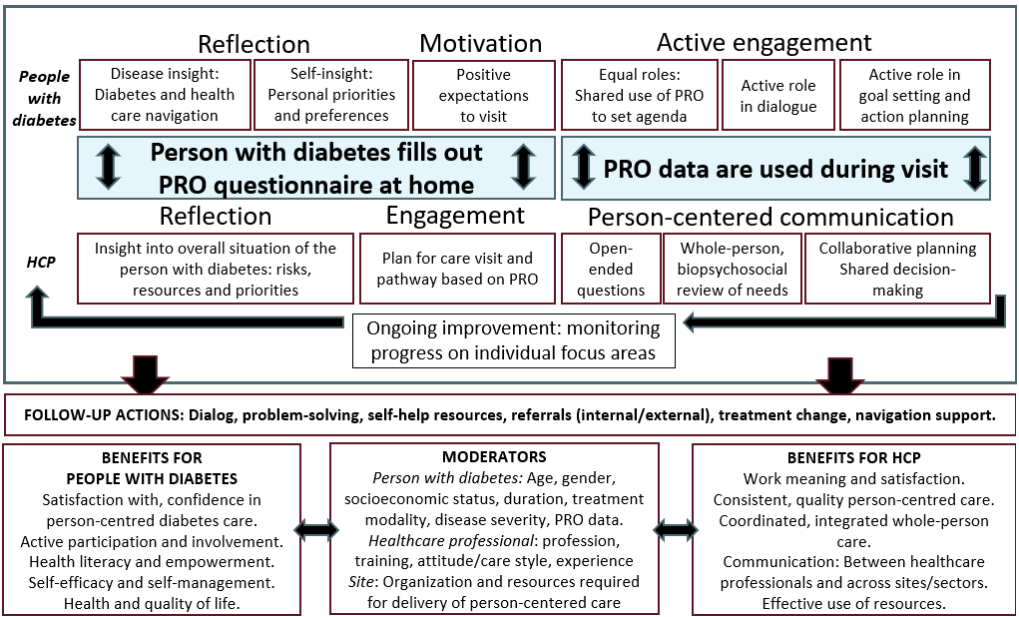
A working model to illustrate hypothesized mechanisms, moderators, and impacts related to the use of the PRO diabetes tool. HCP: health care professional; PRO: patient-reported outcome.

A conceptual model to illustrate the key hypothesized mechanisms of action, moderators, and outcomes for the PRO diabetes intervention is shown in Figure 2. This was used to guide the design of outcomes and data collection tools. The working model reflects preliminary data from the formative evaluation process of the PRO diabetes tool [1,11] and will be continuously updated and expanded with the progress of the study.

The top part of the model illustrates that completion of PRO at home is hypothesized to impact care through mechanisms of reflection, motivation, and engagement before the visit and through mechanisms of active engagement of the people with diabetes and use of person-centered and value-based care strategies by the HCP during the visit. The use of PRO is hypothesized to lead to different follow-up actions by the health care team, which in turn is hypothesized to lead to different benefits for people with diabetes and HCPs. Both patient, health professional, and care setting factors are hypothesized to potentially moderate the extent to which use of the intervention impacts outcomes for people with diabetes and HCPs. Textbox 2 provides an overview of the main outcomes of this study.

**Overview of the main outcomes of the study.**

**Primary outcomes**

- Perceptions of people with diabetes with regard to the following:
  Acceptability, usability, and appropriateness of the patient-reported outcome (PRO) intervention (outcome 1A)
  Impact (positive and negative) on them of using the PRO diabetes tool as part of their care (outcome 1B)
- Perceptions among health care professionals regarding:
  Usability, feasibility, fidelity and appropriateness of the PRO intervention (outcome 2A)
  Impacts (positive and negative) on diabetes care of using the PRO diabetes tool as part of routine care (outcome 2B)

**Secondary outcomes**

- Psychometric and clinical validity and reliability of the PRO questionnaire, scoring algorithms, and clinical dialogue support (outcome 3)
- Barriers and facilitators for the implementation and impact of PRO diabetes tool for people with diabetes, for health care professionals, and at the health system level (outcome 4)
- Estimation of public health impact indicators of the PRO intervention according to the RE-AIM (Reach, Efficacy, Adoption, Implementation and Maintenance) model (outcome 5)

#### Primary Outcomes

This study uses two primary descriptive outcomes. First, data will be gathered on the perceptions of the usability, acceptability, and appropriateness of the PRO diabetes tool of people with diabetes, along with the perceived positive and negative effects of their use of the tool. Data extracted about their disease and care are classified by type of diabetes, age, gender, treatment modality, duration, disease complications, comorbidities, treatment setting, PRO outcomes, and general rating of visit quality (primary outcomes for people with diabetes). In addition, data will be collected on the HCP’s perception of acceptability, appropriateness, and perceived positive and negative effects of the tool on care quality and experience by HCPs. Furthermore, information on years of professional experience, years of diabetes experience, training in use of person-centered communication, confidence in use of PRO in visits, treatment setting, and general rating of visit quality (primary outcome for HCP) is also obtained.

Measures and data sources for outcomes pertaining to indicators of feasibility and implementation [55,56] are detailed in Multimedia Appendix 4.

#### Secondary Outcomes

Characterization of the validity and reliability of the PRO questionnaire and clinical algorithms is a secondary outcome of the study (outcome 3). This outcome is assessed in three ways. First, the people with diabetes’ perception of relevance, comprehensiveness, difficulty, acceptability, appropriateness, and comprehension of the questionnaire according to the type of diabetes, age, gender, treatment modality, duration of diabetes, disease complications, comorbidities, and treatment setting are measured (outcome 3A).

Next, the HCP’s evaluation of the clinical and face validity of the PRO data; the clinical relevance and utility of the PRO content, including the items, scoring, outputs, algorithms, and digital dialogue; and decision support are measured (outcome 3B).

Then, the psychometric properties of the questionnaire pertaining to the extent to which it provides valid, reliable measurements of the selected constructs, can predict relevant future clinical or care needs, events, and prognoses, and can discriminate appropriately between relevant levels of symptom severity are evaluated (outcome 3C).

We also assessed the barriers and facilitators at the people with diabetes, HCP, clinic, and health care system levels for the implementation and impact of the PRO diabetes tool as a secondary outcome (outcome 4). Finally, the initial indicators of the reach, efficacy, adoption, implementation, and maintenance of the intervention in accordance with the RE-AIM model will be examined (outcome 5). The measures and data sources used to evaluate the intervention according to RE-AIM indicators are detailed in Multimedia Appendix 5.

### Participant Timeline

**Figure 3:**
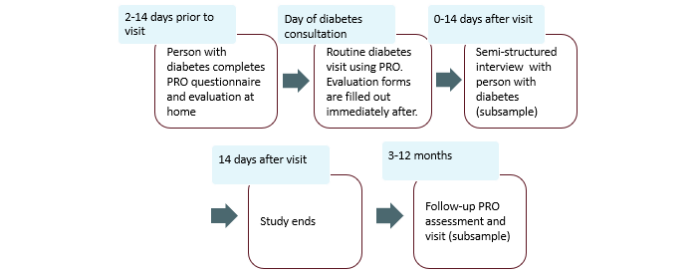
Timeline for people with diabetes participating in the study. PRO: patient-reported outcome.

**Figure 4:**
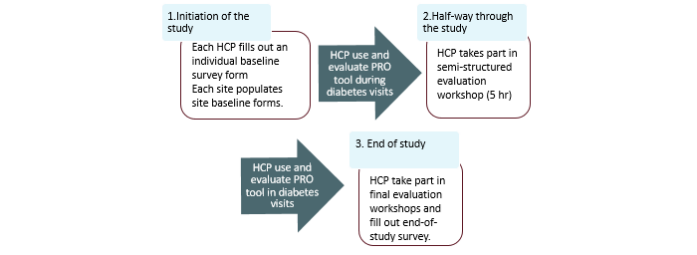
Timeline for HCPs participating in the study. HCP: health care professional; PRO: patient-reported outcome.

All sites used the same core data collection and intervention procedure, as shown in Figures 3 and 4, with local modifications and adaptations, for seamless integration into routine care. Each participant was recruited approximately 14 days before their scheduled visit. People with diabetes will receive a link or electronic invitation to access and complete informed consent and the PRO Diabetes Questionnaire 2-14 days before their visit. People with diabetes will complete the PRO before their visit, unless there are specific barriers preventing this. At their scheduled visit, the HCPs use the IT PRO Dialogue Tool to review the results with the people with diabetes to support their dialogue. People with diabetes and HCPs will independently complete the evaluation forms when they are physically separated after the visit. Selected people with diabetes are invited for a 30- to 45-minute semistructured interview within 0-12 days after their diabetes visit. HCPs will participate in 4- to 5-hour multidisciplinary, structured HCP evaluation workshops halfway through and at the end of the study as well as complete individual end-of-study questionnaires.

### Sample Size

As the study has descriptive purposes, formal requirements for sample size estimation for the primary outcome were not applied. However, for any subgroup analysis, the minimum number of patients per group was estimated to be 64. For continuous variables, a minimal subgroup size of 64 was estimated to achieve 80% power for detecting an effect size of 0.5 at a significance level of *P*=.05 by using a two-sided, 2-sample, equal-variance *t* test. For proportions, minimal group sample sizes were estimated to be 63 to achieve 80% power for detecting an effect size of 0.5 with a significance level of .05 by using a two-sided z test in a similar manner.

The minimum target for recruitment was 125 people with type 1 diabetes and 375 people with type 2 diabetes, based on requirements for comparatively analyzing by subgroups and requirements for psychometric analyses.

According to the available data, group comparisons will be undertaken using data across all centers by age groups, treatment modality (none, tablet only, short- and long-acting insulin, insulin pump, and glucagon-like peptide-1), blood sugar measurement technology (finger prick, flash glucose monitoring, and continuous glucose monitoring), diabetes complications (neuropathy, cardiovascular disease, gastrointestinal complications, sexual dysfunction, and sleep difficulty), and comorbidities.

Power analyses for comparative analyses will be conducted based on the final number of people with diabetes by site for PRO and PRO evaluation data to determine the extent to which outcomes can be comparatively analyzed either at the site or at the care-setting level.

The expected number of HCPs to participate was 25-40, based on what was reported to be feasible by the study sites. Sites were encouraged to have several HCPs participating as a minimum and to include HCPs with a good diversity of health care discipline, age, diabetes experience, and profession.

To compare experiences across different health care settings, the aim of this study was to include sites from secondary care, municipality rehabilitation centers, and primary care. On the basis of the resources available for the pilot study, the total number of sites was estimated to be 7-10.

### Recruitment

During the recruitment period from November 2019 to December 2020, each site recruited people with diabetes who met the eligibility criteria as part of their routine practice. The sites molded the recruitment procedures based on their local care flow and requirements for the use of PRO in routine diabetes care visits or rehabilitation. At every site, recruitment involves inviting eligible people with diabetes who are registered or scheduled for a diabetes care visit to try the PRO diabetes tool in conjunction with their upcoming visit. Only people with diabetes who provided written informed consent for participation in the study are included. Each site uses different methods of communication with people with diabetes for recruitment depending on their routine care pathways, including using the phone, electronic (app or email), and in-person invitations.

### Data Collection

#### Overview

The data collection and data collection tools are listed in Textbox 3. Data for the primary outcomes are collected using Likert scales and open-ended evaluation questionnaires that are completed by people with diabetes and HCPs in connection with the use of the PRO diabetes tool in routine care and by HCPs at the end of the study. In the mixed methods analysis, qualitative data are collected from transcribed interviews, consultations, evaluations, and debriefing workshops completed during the entire study period.

**Data collection tools. The contents of the main data collection tools are available in the multimedia appendices.**

**Multicenter patient-reported outcome (PRO) diabetes study** **core data collection tools**

- People with diabetes
  - PRO Diabetes Questionnaire (Multimedia Appendix 6)
  - PRO Evaluation Questionnaires (PRO-EVAL-P; Multimedia Appendix 7)
  - Post-Visit PRO Evaluation Questionnaire (PRO-CON-EVAL-P-SF; Multimedia Appendix 8)
  - Semistructured interview guide for people with diabetes (Multimedia Appendix 9)
  - Baseline sociodemographic data sheet for people with diabetes (Multimedia Appendix 10)
- Health care professional (HCP)
  - Baseline background data (HCP profile questionnaire; Multimedia Appendix 11)
  - Post-Visit PRO Evaluation Questionnaire (PRO-CON-EVAL-HCP-SF) and evaluation form for algorithm evaluation (Multimedia Appendix 12)
  - Semistructured guide for HCP evaluation workshops (HCP evaluation guide; Multimedia Appendix 13)
  - HCP End-of-Study Evaluation (HCP end-of-study PRO evaluation form; Multimedia Appendix 1)
- Study site
  - Diabetes Clinic Resources for Person-Centered Diabetes Care Survey (Multimedia Appendix 14 [57])
  - Site datasheet: Organization, services, population, resources for PRO follow-up

#### Data Collection for Primary Outcome 1: People With Diabetes’ Perceptions of the PRO Diabetes Questionnaire

The perceptions of people with diabetes regarding the PRO Diabetes Questionnaire are evaluated by the people with diabetes at home immediately after completing it using a digital PRO Evaluation Questionnaire (PRO-EVAL-P). This immediate evaluation of the PRO Diabetes Questionnaire by the people with diabetes assesses the perceived relevance of the questionnaire in a realistic scenario before their routinely scheduled visit and allows for both closed and open-ended responses.

#### Data Collection for Primary Outcome 1: People With Diabetes’ Perceptions of the Dialogue and Impact of the PRO Diabetes Questionnaire

This outcome assesses the multi-item and single-item scores from the people with diabetes evaluations using the Post-Visit PRO Tool Evaluation Questionnaire after each visit where the PRO tool was used (PRO diabetes visit). To minimize bias due to social desirability, people with diabetes are informed that their evaluation will be kept confidential, that their HCP will not see their responses, and that their answers will only be used in research after deidentification. These data provide important new information regarding the diversity and variability of individual experiences of people with diabetes when using the PRO diabetes tool in the visit and allow for an analysis of interactions among HCPs, people with diabetes, and setting factors in relation to primary outcomes. Verbatim transcripts of structured interviews conducted with a subset of a minimum of 10 randomly selected people with diabetes in each care setting after their PRO diabetes visit are coded and analyzed for mixed methods analysis.

#### Data Collection for Primary Outcome 2: HCP’s Perceptions of the Use of the PRO Diabetes Tool in Individual Care Visits

These outcome data were obtained from the multi-item and single-item scores for each PRO visit in the study using the HCP evaluations on the Post-Visit PRO Tool Evaluation Questionnaire immediately after or within a few days of the visit. The HCPs will complete informed consent and are informed that the collected data are only used in an anonymized manner. In addition, the HCPs will complete evaluation questionnaires at the end of the study, which evaluate their overall views and attitudes regarding the use of PRO, impact of use of PRO, and requirements for use of PRO.

#### Data Collection for Secondary Outcomes: Predictors, Barriers, Facilitators, and Readiness Factors for the Adoption, Usage, Satisfaction, and Benefits of PRO

Sociodemographic and diabetes profile data were collected for all participating people with diabetes to analyze the known-group and discriminative validity and reliability of the PRO Diabetes Questionnaire together with primary study outcomes by people with diabetes subgroups. A core set of HCP profile data is collected for all HCPs to evaluate primary outcomes by the relevant HCP subgroups. Two comprehensive questionnaires are used—one adapted for use in Denmark for this study from the Primary Care Resources for Chronic Illness Care Questionnaire [57] (Multimedia Appendix 14) and one study-specific questionnaire. The study-specific questionnaire assesses details for each site about the local PRO setup and examines the extent to which the site has resources and services to follow-up on each PRO construct covered by the PRO Diabetes Questionnaire. These data are used to qualitatively analyze relationships between primary outcomes by contextual factors relevant to the delivery of person-centered and psychosocial diabetes care and the RE-AIM analysis.

#### Likert Scale Evaluation Questionnaires of Experience of Use of PRO in Routine Care

We did not identify any previously published PRO evaluation questionnaires that had been specifically designed and validated for our intended large-scale use in routine care settings. The VBHC-PRO-DIA team therefore developed and tested a set of purpose-built Likert Scale evaluation questionnaires in collaboration with people with diabetes and HCPs as part of the PRO tool’s formative evaluation [29]. A first set of long form questionnaires was developed for a hospital-based clinical study to evaluate the impact of the PRO diabetes tool on person-centered communication and autonomy support using multi-item scale scores.

For this study, HCPs from all care settings and a panel of people with diabetes were involved to identify a core set of evaluation questions that were appropriate and acceptable for use in all sites and care settings, which would allow for pooled and comparative analyses. The short-form questionnaires evaluated experiences of people with diabetes and HCPs related to the use of PRO data in their diabetes visit, such as the extent to which the data were used, impact on engagement, dialogue and person-centered care qualities of the visit, impact on self-management and on care quality, and overall satisfaction and interest in continued use. The questionnaires also briefly assess the perceptions of people with diabetes and HCPs on the general quality of the dialogue and aspects of person-centered communication [10,58].

### Data Management

Each research site is responsible for data entry, security, storage, and data quality verification. Of the 10 study sites, 9 opted to use a uniform web-based research data collection system that was purpose-built for M-PRODIA data collection by Aalborg University Hospital. The web-based data collection forms for the M-PRODIA are integrated into the DiaProfil solution for seamless completion by both people with diabetes and HCPs as part of the study procedures. In the hospital study site in the Capital Region, where a separate health platform system is used for PRO data collection and dialogue support, evaluation forms are provided in paper and pencil format, and manual data entry is performed by clinical staff using formats that are comparable with the electronic forms. Double-entry is used for the sample cases. All paper forms and raw data will be stored for 5 years or as required by law.

A comprehensive codebook with operational definitions, numerical codes, and standardized formats for all variables was established for data monitoring, quality control, coding, and harmonization purposes. At multiple points during the study period, sample data are collected to assess data completeness, missing value patterns, and range checks. At the halfway point, the HCP will participate in an interim study review meeting where data quality is evaluated.

### Data Analysis

#### Statistical Methods

Quantitative data (ie, closed-ended questionnaire data and numerical data) will be coded, and all numerical data will be prepared for descriptive analyses and main statistical analyses using IBM SPSS Statistics for Windows (IBM Cooperation). The SAS statistical software package (SAS Institute Inc) is used for multilevel hierarchical regression analyses. Descriptive statistics will be used for continuous (mean and SD) and categorical (frequency and percentage) variables to analyze and compare evaluation data by center and subgroups. The aim of this study is to analyze drop-out data, including nonparticipation, attrition, and completion rates across centers, to characterize the generalizability of findings relating to population reach and outreach to vulnerable populations, and to compare implementation drivers across participating centers. Both parametric and nonparametric statistics were used for group comparisons based on the distribution of each data variable.

Missing value analysis, including the identification of variables missing completely at random or not at random, will be conducted for all variables.

PRO results are scored and analyzed as both raw and scored data using predefined clinical scoring algorithms according to subgroups of adequate sizes for multilevel regression and multilevel logistic regression models [59]. Multi-item scores will be transformed and standardized to a score range of 0-100.

For subgroup comparisons, two-tailed *t* tests and nonparametric statistics, such as the Mann-Whitney *U* test, are used. Multivariate mediation regression analysis will be used to examine the relative contribution of the different participant, treatment, and setting characteristics. Multilevel analyses will be used as feasible and required to separate the variance by people with diabetes, HCP, and care setting. Bonferroni and associated methods will be used to adjust P values for multiple comparisons. Intraclass coefficient analysis will be used to examine the level of agreement between HCPs and people with diabetes on the primary outcomes of the visits, which were evaluated both by people with diabetes and HCPs.

#### Psychometric Analysis

Study variables will be evaluated for skewed or nonnormal distributions using measures of dispersion (eg, means and SDs, kurtosis, and Pearson skew coefficients). Cosmin guidelines are used as a reference framework for the characterization of psychometric reliability and validity [60,61]. Exploratory and confirmatory factor analyses will be used to examine the hypothesized multi-item scales for multi-dimensionality, convergent validity, and discriminant validity. Internal consistency of the multi-item scales will be examined using accepted methods, including Cronbach α or Kuder-Richardson reliability coefficients, as appropriate. We will use Pearson product-moment and Spearman correlation coefficients, depending on the nature of the data, to assess the construct validity between PROs and hypothesized validity variables (eg, glycemic control and use of health services). If feasible, we will estimate the responsiveness of PRO indicators to change and minimal clinically important differences where follow-up PRO data are available.

#### Qualitative Analysis

Qualitative data (eg, focus groups, workshops, interview transcripts, open-ended responses from evaluation questionnaires, notes, minutes, and video, audio, and graphic outputs from evaluation and study activities) were entered and coded in NVivo 12 (QSR International).

Multiple qualitative analysis methods will be used for different purposes and, as relevant, for specific qualitative data [62-64]. Qualitative data are primarily coded and categorized using content and semantic analysis, and where appropriate, using mixed inductive-deductive analysis rooted in a phenomenological approach [65] with the involvement of multiple coders.

A code book using the taxonomy of the PRO questionnaire content (ie, items and constructs) is used across data from all informants to examine the validity, utility, and perceived benefits and enable multi-informant triangulation analysis for each individual PRO construct and item.

Qualitative data pertaining to implementation from HCP evaluation workshops and center evaluations will be analyzed with guidance from the RE-AIM framework [64].

In this way, data can be combined to evaluate indicators of the PRO questionnaire relevance, acceptability, difficulty, comprehensiveness, comprehension, clinical utility, validity, scoring validity, self-insight, utility, emotional impact, and emergent insights. To the extent feasible, coding will also cover indicators of fidelity, feasibility, appropriateness, care quality impact, engagement, benefits, disadvantages, barriers and facilitators, attitudes, sustainability, and emergent insights.

#### Mixed Methods Analysis

We aim to combine qualitative and quantitative data from different informants (ie, people with diabetes, HCPs, and centers) using convergent and parallel mixed methods research designs to provide increased robustness and depth of analyses [66]. An explanatory mixed methods analysis approach is completed using the coding of transcribed interviews of people with diabetes. Where feasible and appropriate, the frequency of codes will be used to supplement and corroborate the quantitative analyses of primary outcomes and to characterize any differences across subgroups [67,68].

#### Data Monitoring

Each participating site collects and is responsible for its own data and the delivery of these data to the centralized VBS-PRO-DIA research group at the Department of Endocrinology at Aalborg University Hospital, Aalborg, Denmark, in accordance with specified requirements for the anonymization of data, use of study-specific IDs, and secure data transfers. Furthermore, each site was responsible for ensuring that informed consent was obtained and validated for all participants. All primary analyses were single-arm and noncomparative. Data from each participating site were traceable in the analyses. A data monitoring committee was not deemed necessary because of the accessibility of the data and the nature of the study as a real-world pilot study evaluation.

#### Interim Analyses and Stopping Guidelines

We conducted an interim analysis at the halfway point of the study in 2020 to assess the recruitment status and analyze a sample of interim results to consider potential early termination of the study. The study is considered to carry minimal risk for the participants, as the PRO Diabetes Questionnaire had been developed with and evaluated by people with diabetes in advance.

Evaluation questionnaires used from the beginning of the study period after each diabetes visit included specific prompting questions for the people with diabetes to determine if there were any unpleasant experiences or problems related to using the PRO Diabetes Questionnaire. Specifically, we wanted to understand if, due to patient characteristics or treatment context, certain questions, such as those relating to sexual dysfunction, loneliness, or psychological well-being, would be experienced by some people with diabetes as unpleasant. Alongside the ongoing communication with HCPs involved in the use of PRO, these questionnaires ensure ongoing continuous monitoring for potential negative effects of the intervention. Interviews were conducted with approximately 5 people with diabetes from each site halfway to obtain qualitative insights regarding any positive and negative experiences associated with the study at each site.

#### Auditing and Independent Data Verification

The Danish National Health Data Authorities independently reviewed the transcripts of the evaluation interviews with people with diabetes, verbatim evaluation of the open-ended responses from the HCPs and people with diabetes following each visit, and the transcripts of the HCP evaluation workshops for vetting and auditing analysis.

### Ethics and Dissemination

#### Research Ethics Approval

The M-PRODIA has been approved by the regional research approval authority and evaluated by the authority not to be in the scope of the scientific ethics committee evaluation as the study does not involve the collection of human biological materials and does not involve medical intervention.

#### Protocol Amendments

This is a pragmatic and formative real-world pilot study, so insights and data obtained during the study period may be used to adjust and optimize the study protocol.

#### Informed Consent

Each study site is responsible for obtaining informed consent from all people with diabetes and HCPs participating in the study. Consent forms are locally adjusted if necessary, in accordance with the specific data that are collected from each site. A duly signed and complete informed consent form is a prerequisite for transferring anonymized data to the M-PRODIA research team.

#### Confidentiality

Anonymized, deidentified data using study IDs for people with diabetes, HCPs, and evaluated visits are sent from each site using secure data transmission to the M-PRODIA research team at Aalborg University Hospital. Data are stored and processed using data protection procedures in accordance with the requirements of the Danish Data Protection Agency and the region of northern Denmark.

#### Dissemination Policy

Results will be disseminated to guide implementation of the PRO diabetes tool in the national health system in collaboration with the Danish National Health Data Authority and health care stakeholders. Vancouver guiding rules for authorships will be used for scientific publications [69]. Interim findings will be shared with the Danish Health Data Authority in support of the public health objectives of the national pilot study.

## Results

A total of 7 study sites, 598 people with diabetes, and 34 HCPs completed the study by April 1, 2021. Data cleaning and management are ongoing. Primary results based on primary outcomes are expected to be disseminated by the end of 2021.

## Discussion

### Study Implications

This is the first large-scale study to evaluate the use of the national PRO diabetes tool in routine care across different health care settings using psychometrically tested PRO evaluation questionnaires for both people with diabetes and HCPs.

This study is expected to provide important new information about perceived acceptability, relevance, and benefits of the PRO diabetes tool in a large representative and heterogeneous population of adults with type 1 and 2 diabetes in Denmark and in a diverse group of HCPs representing multiple professions and different health care settings.

Although a great deal of research has focused on evaluating the psychometric characteristics of PRO diabetes questionnaires [70-72], we found very limited research examining how people with diabetes perceive the relevance and personal value of a comprehensive digital PRO questionnaire in the context of their routine diabetes care.

We believe this study generates important new insights into the experiences of using PRO in diabetes care, which can contribute to the identification of strategies for improvement of the questionnaire, its use, and implementation and provide general insights to guide future digital PRO interventions.

This study is the result of a long-standing national multidisciplinary and patient-centric collaboration to design and evaluate a nationally agreed PRO diabetes tool. It is hoped that the implementation of a shared national PRO diabetes tool, created with the involvement of all health sectors, can help facilitate a coordinated, continuous person-centered care experience for people with diabetes in Denmark over time. However, this study reflects that we are only at the beginning of a long learning curve. Long-term implementation, health and cost-effectiveness research, and collaborative quality assurance efforts are warranted to optimize and evaluate the long-term impact of implementation across the health system and communities. The introduction of this PRO tool on a larger scale may strengthen the role and influence of people with diabetes and family members of people with diabetes in the health care system in multiple ways, which may change how people with diabetes and family members of people with diabetes communicate about diabetes, how HCPs communicate within and across teams about person-centered care, and how health care sectors and communities communicate and coordinate about the provision of person-centered diabetes care.

### Methodological Considerations

The main strengths of this study are that it combines qualitative and quantitative data to provide detailed insights into the perspective of people with diabetes on the real-world use of PRO and provides a wide range of insights regarding the many factors likely to influence the public health impact potential of the PRO diabetes tool in Denmark.

This pilot study has important limitations. One limitation is the lack of a randomized control group and the other is that the main outcomes are mainly process-orientated and rely solely on self-reported data, which may be subject to bias related to social desirability and recall issues.

The study is not designed to quantify the magnitude of clinical, health, and empowerment-related benefits that may be achieved by using the PRO intervention. The study also does not compare reach and effectiveness performance of different PRO tools, which will be important to determine mechanisms of action. These are important research questions that must be addressed in parallel and planned research. Separate clinical studies by the VBHC-PRO-DIA team are underway to address these issues separately and to examine the impacts of PRO use over time on clinical and health care use indicators and health cost drivers.

### Study Status

The main fieldwork and recruitment of people with diabetes and HCPs for the M-PRODIA was completed in January 2021; however, due to the challenges and restrictions imposed by COVID-19, some study sites have encountered delays in data collection. Therefore, the closure of the database was expected by April 2021.

### Conclusions

A detailed study protocol and newly developed psychometrically designed data collection tools are developed and implemented in 2020 to collect detailed data on how people with diabetes and HCPs experience the use of a newly designed digital PRO tool in routine diabetes care across different care settings.

## Appendix

Multimedia Appendix 1 Health care professional guidance for the use of patient-reported outcomes in annual outpatient diabetes visits.

Multimedia Appendix 2 DiaProfil patient-reported outcomes dashboard screenshot.

Multimedia Appendix 3 Feasibility and implementation indicators and measurements.

Multimedia Appendix 4 Indicators and measurements used for the Reach, Efficacy, Adoption, Implementation and Maintenance evaluation.

Multimedia Appendix 5 Content of the Danish Patient-Reported Outcomes Diabetes Questionnaire.

Multimedia Appendix 6 Patient Evaluation of the Patient-Reported Outcomes Questionnaire (PRO-EVAL-P).

Multimedia Appendix 7 Patient evaluation of patient-reported outcomes use after the diabetes visit (PRO-CON-EVAL-P-Short Form).

Multimedia Appendix 8 Semistructured interview guide for use with people with diabetes.

Multimedia Appendix 9 Background and sociodemographic data for people with diabetes.

Multimedia Appendix 10 Health care professional Baseline Profile Questionnaire.

Multimedia Appendix 11 Health care professional evaluation of the use of patient-reported outcomes (PRO-CON-EVAL-HCP in algorithm review form).

Multimedia Appendix 12 Semistructured guide for health care professional evaluation.

Multimedia Appendix 13 Health care professional End-of-Study Patient-Reported Outcomes Evaluation Questionnaire.

Multimedia Appendix 14 Center Resource Profile for Person-Centered Diabetes Care.

## Abbreviations

HCP: health care professional
M-PRODIA: multicenter patient-reported outcome diabetes study
PRO: patient-reported outcome
RE-AIM: Reach, Efficacy, Adoption, Implementation and Maintenance
VBHC-PRO-DIA: Value Based Health Care and PRO in Diabetes Project
