# Supplementary material for: Perceived Benefits, Barriers, and Facilitators of a Digital Patient-Reported Outcomes Tool for Routine Diabetes Care: Protocol for a National, Multicenter, Mixed Methods Implementation Study
Source: JMIR Res Protoc. 2021 Sep 3;10(9):e28391. doi: 10.2196/28391 (PMC8449301; doi:10.2196/28391)
Supplement: Multimedia Appendix 1 [file resprot_v10i9e28391_app1.docx]

**Multimedia Appendix 1.** Health care professional guidance for the use of patient-reported outcomes in annual outpatient diabetes visits. Guidance for HCP: How to use the PRO dashboard (DiaProfil) during a clinical diabetes outpatient visit.

**Before the visit:**

- HCP opens DiaProfil PRO dashboard on a PC.
- HCP reviews PWD’s PRO results on equal terms as other relevant health, treatment and test result (e.g., eye screening, blood test results) information.
- The PC screen is set so that the dashboard can be seen equally well by HCP and PWD and eye contact can be maintained throughout the conversation.

**The visit:**

Visit is commenced and it is stated that PRO data is available:

- *“Thank you for answering the questionnaire…”*
- *"How did you experience answering the questionnaire?"*
- *"The purpose of your questionnaire response is to include it in our conversation in order to set the framework for the topics of the consultation.”*

The PRO display is introduced:

Briefly explain the PRO dashboard to the PWD with focus on the green/yellow/red colors and the location of PWD’s requested discussion topics on the screen.

Check with the PWD if the answers regarding foot and eye screening the past year are correct.

Use open-ended questions to obtain more information regarding relevant PRO areas, e.g:

- *"Can you recognize these answers from your daily life routine with diabetes?"*
- *"Has anything changed since you answered the questionnaire?"*

Common agenda:

Establish a common agenda using PRO, clinical data, and additional input from the PWD. Prioritize the topics and the order of the topics together with the PWD. E.g.:

- *“What hopes/goals do you have for this consultation today?”*
- *"I see that you answered in the questionnaire you would like to discuss these topics today…"*
- *"Are there topics - in addition to your answer - that you would like to discuss today?"*
- *“When I read your answers, I especially noticed that…. which I think we should discuss further”*

Review or consider any relevant PRO, lab test results, blood sugar monitoring data. Renew prescriptions and update chart data.

Summarize conclusions and agreements together with PWD:

- *"Today we have discussed several different areas in relation to your daily life with diabetes such as…"*
- *“What is most important for you to focus on in the future?”*
- *“How can I (the HCP team) help you achieve that?*

Follow-up on relevant actions, referrals or educational resources identified using DiaProfil during the visit.

**In case of cancellation, illness or no-show of the PWD:**

The HCP is responsible for reviewing the PWD’s PRO data. If it is not feasible to do in a consultation, the HCP is responsible for responding to PWD’s answers either by letter or telephone.

This is a Multimedia Appendix to a full manuscript published in the JMIR Research Protocols. For full copyright and citation information see <http://dx.doi.org/10.2196/jmir.28391>.

Developed by Aalborg University Hospital, Denmark, 2019
