# Supplementary material for: Perceived Benefits, Barriers, and Facilitators of a Digital Patient-Reported Outcomes Tool for Routine Diabetes Care: Protocol for a National, Multicenter, Mixed Methods Implementation Study
Source: JMIR Res Protoc. 2021 Sep 3;10(9):e28391. doi: 10.2196/28391 (PMC8449301; doi:10.2196/28391)
Supplement: Multimedia Appendix 2 [file resprot_v10i9e28391_app2.docx]

**Multimedia appendix 2:**
DiaProfil PRO Dashboard Screenshot


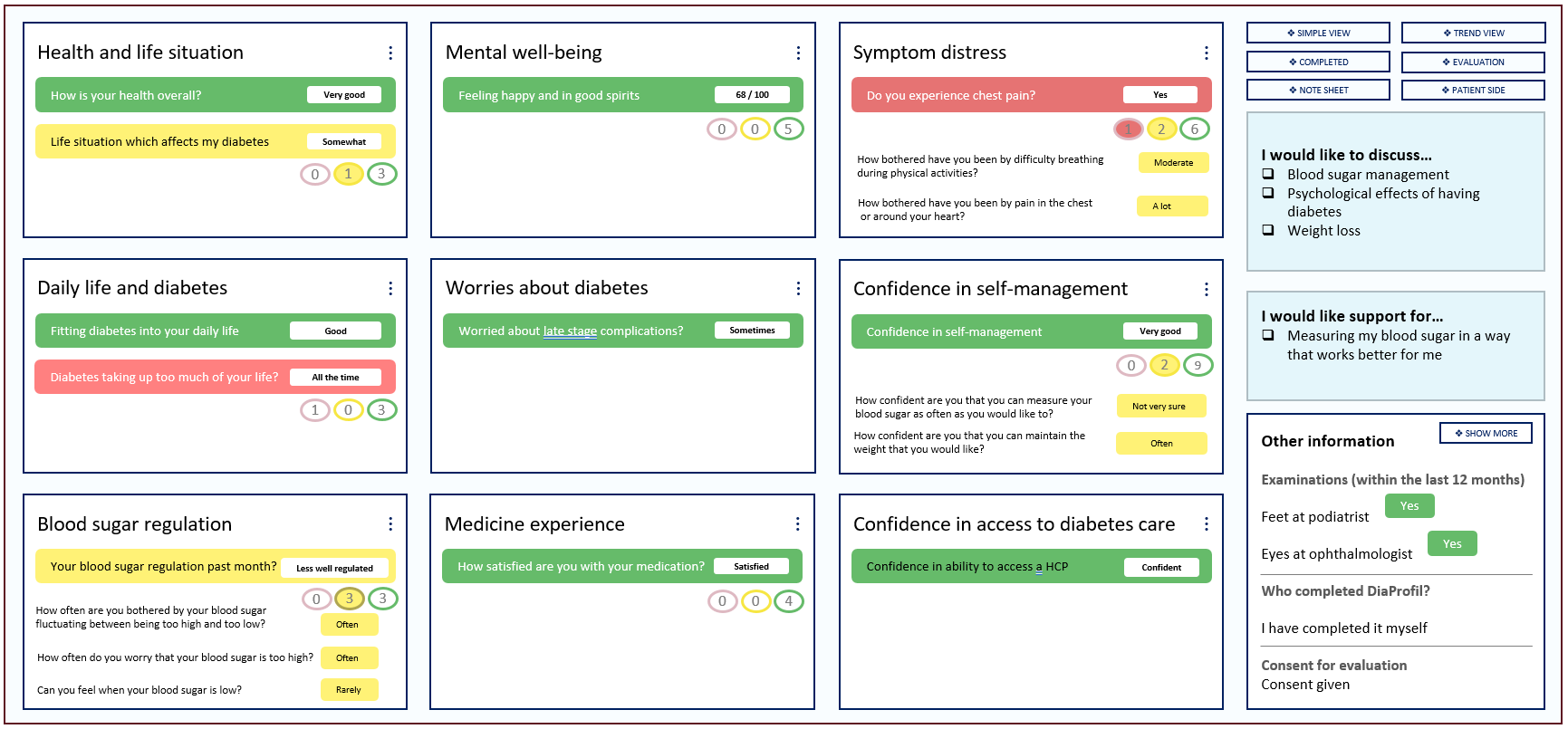


A graphic illustration of the elements in a Diaprofil Dashboard. Each of the nine squares represent a topic category from the PRO diabetes questionnaire. The screen provides a one-glance overview of the level of potential problem areas. The right column shows the categories, or the open-ended response provided by the PWD to set the specific agenda for their consultation with their HCP.

This is a Multimedia Appendix to a full manuscript published in the JMIR Research Protocols. For full copyright and citation information see <http://dx.doi.org/10.2196/jmir.28391>.

Developed by Aalborg University Hospital, Denmark, 2019.
