# Supplementary material for: Perceived Benefits, Barriers, and Facilitators of a Digital Patient-Reported Outcomes Tool for Routine Diabetes Care: Protocol for a National, Multicenter, Mixed Methods Implementation Study
Source: JMIR Res Protoc. 2021 Sep 3;10(9):e28391. doi: 10.2196/28391 (PMC8449301; doi:10.2196/28391)
Supplement: Multimedia Appendix 3 [file resprot_v10i9e28391_app3.docx]

**Multimedia appendix 3:**Feasibility and implementation indicators and measurements

| **Feasibility and Implementation Indicators** | **Measures** | **Data Sources** |
| --- | --- | --- |
| Acceptability – by all relevant stakeholders for diabetes care | - Percentage of PWD reporting a positive influence related to the use of the PRO diabetes Questionnaire - Percentage of HCPs reporting that the PRO Diabetes Tool was value-adding and relevant for continued use. | - Post-visit evaluation questionnaires - Post-visit semi-structured patient interviews - HCP semi-structured evaluation workshops - Multi-stakeholder national work group - Selected sites: Visit observations/recordings |
| Appropriateness | - Percentage of PWD finding the tool relevant (by setting, visit type, patient profile). - Percentage of HCPs finding the tool appropriate (by setting, visit type, profession). - Utility of PRO output for screening, dialogue- and decision-support, treatment and outcome monitoring and potential for use during pre-visit visitation support. | - Post-visit PWD and HCP evaluations - Post-visit PWD interviews - HCP evaluation workshops - Multi-stakeholder national working group - Selected sites: Parallel pre-post clinical study impacts on care activities/resource use. Observations/recordings of visits. |
| Feasibility | - Practicality and feasibility of using the PRO tool in routine practice with existing, available resources | - HCP evaluation workshops - HCP interviews - HCP and PWD post-visit evaluations |
| Fidelity | - Percentage of the PRO is completed at home prior to visit - Percentage of visits where the PRO is used as intended - Percentage of HCP who use the PRO to prepare for visit - Percentage using the shared dashboard during visits - Percentage using the tool to identify follow-up action | - Post-visit evaluation questionnaires - Post-visit semi-structured patient interviews - HCP evaluation workshops |
| Implementation Cost | - Identify cost drivers for the intervention delivery - Identify cost drivers for implementing a delivery strategy | - Staff interviews (if/as feasible) - Administrative data (if/as feasible) |

Overview of key indicators identified as relevant to evaluate feasibility of data collection for pertaining to acceptability, appropriateness, feasibility, fidelity and implementation cost.

This is a Multimedia Appendix to a full manuscript published in the JMIR Research Protocols. For full copyright and citation information see <http://dx.doi.org/10.2196/jmir.28391>.

Developed by Aalborg University Hospital, Denmark, 2019.
