# Supplementary material for: Perceived Benefits, Barriers, and Facilitators of a Digital Patient-Reported Outcomes Tool for Routine Diabetes Care: Protocol for a National, Multicenter, Mixed Methods Implementation Study
Source: JMIR Res Protoc. 2021 Sep 3;10(9):e28391. doi: 10.2196/28391 (PMC8449301; doi:10.2196/28391)
Supplement: Multimedia Appendix 4 [file resprot_v10i9e28391_app4.docx]

**Multimedia appendix 4:**Indicators and measurements for RE-AIM evaluation

| **RE-AIM Indicators** | **Measures** | **Data sources** (Not all data sources are applicable to all sites) |
| --- | --- | --- |
| **Reach** | Proportion of eligible and high-need target populations:   - 1. Offered to use the PRO   2. Accepting of the PRO   3. Completing the PRO questionnaire   4. Completing the PRO diabetes consultation as planned   Factors indicative of accessibility for the full population, hereunder vulnerable and hard-to-reach subgroups. | Estimates by pilot sites from the available information derived from routine pilot testing. Depending on local site set-up:   - Recruitment logs - HCP registration sheets - Exit questionnaires - Administrative data analysis - PRO data registration - PRO dashboard and visit use registration |
| **Efficacy** | - Preparation and active engagement of the PWD - Quality of the dialog and care visit - Care/education quality, follow-up, and actions - Self-care, health, and PWD relevant outcomes - Self-care, clinical and PRO outcomes and health care provision and utilization - Exploratory: Benefits expressed by PWD and HCP | Indicators of perceived efficacy and effects by PWD and HCP:   - Post-visit evaluation questionnaires - Post-visit semi-structured patient interviews - HCP Evaluation focus groups and workshops   Selected sites: Pre-post clinical chart data, administrative and activity monitoring |
| **Adoption** | Proportion of HCPs and practices:   1. Invited to use the PRO Diabetes Tool 2. Accepting of the PRO Diabetes Tool   3) Using the PRO Diabetes Tool during visits | - Administrative data - PWD post-visit questionnaires (fidelity) - HCP evaluation questionnaires - IT system meta-data on PRO diabetes usage |
| **Implementation** | Proportion of visits where:   1. HCPs actively use the PRO results in visits (levels of use) 2. HCPs use a shared screen/PRO dashboard review with PWD 3. HCPs who apply the PRO systematically in care processes and planning   Proportion of PWD who receive feedback regarding their PRO data from their HCP | - Post-visit patient evaluation questionnaires - Post-visit HCP evaluation questionnaires - HCP evaluation interviews and workshops - IT meta-data on PRO diabetes tool/data usage - Select sites: Observations and consultation recordings. |
| **Maintenance** | - Proportion of HCPs interested in continuing to use the PRO tool. - Proportion of sites interested in continuing to use the PRO tool - Proportion of sites continuing to use PRO tool (resourced, planned) - PRO integration into care pathways, workflow, and IT systems - Adaptation of use of PRO to fit shifting local needs (e.g., virtual care consultations, projects for outreach to specific populations) - Integration of the PRO in strategies for future care with a realistic budget - HCP training/competency plans for onboarding HCPs to use the PRO - Resources and activities for ongoing quality assurance of the PRO use - Resources and activities for ongoing input from PWD about using the PRO | - End-of-study HCP evaluation questionnaire - HCP evaluation workshops - Post-visit evaluation questionnaires (HCP/PWD) - HCP evaluation interviews and workshops - User panel meetings, assessments and evaluations - Administrative desk research and data - Post study interviews and follow-up meetings. |

This is a Multimedia Appendix to a full manuscript published in the JMIR Research Protocols. For full copyright and citation information see <http://dx.doi.org/10.2196/jmir.28391>.

Developed by Aalborg University Hospital, Denmark, 2019.
