# Supplementary material for: Perceived Benefits, Barriers, and Facilitators of a Digital Patient-Reported Outcomes Tool for Routine Diabetes Care: Protocol for a National, Multicenter, Mixed Methods Implementation Study
Source: JMIR Res Protoc. 2021 Sep 3;10(9):e28391. doi: 10.2196/28391 (PMC8449301; doi:10.2196/28391)
Supplement: Multimedia Appendix 5 [file resprot_v10i9e28391_app5.docx]

**Multimedia appendix 5:**Content overview of the pilot version of the Danish PRO diabetes questionnaire (2020).

| Danish PRO diabetes content outline (v1.0 2020) | |
| --- | --- |
| **PRO construct** | **Description (item content)** |
| **1. Self-reported general health** | - How do you rate your overall health? |
| **2. Psychological well-being** | How often… - Cheerful and good spirits - Calm, relaxed - Active and vigorous - Woke up fresh and rested - Life filled with things that interest me |
| **3. Depression** | How often - Felt sad - Lacked interest in daily activities |
| **4. Social support** | - Does it happen you are alone when you would prefer to be around others?  - Do you have someone to talk to if you have problems or need support? |
| **5. Life issues affecting diabetes management** | Do you currently have challenges or problems in life, which make it difficult to manage your diabetes? (e.g., in relation to family, work, finances, or other health problems). (VBHC-PRO-DIA) |
| **6. Daily life with Diabetes** | - How is it going fitting diabetes into your life? |
| **7. Diabetes related distress and negative impacts** | How often  - Does your diabetes take up too much of your daily life?  - Do you worry about diabetes complications? - Does your diabetes prevent you from doing the things that you want? Conditional on response to 3): - limit your activities with family, friends and other people? - Make it more difficult to do your work, study or hobbies? - Make it more difficult to do you your ordinary daily? |
| **9. Diabetes-specific social support** | I have family, friends, or other people with whom I can share my thoughts and feelings about diabetes |
| **10. Confidence in diabetes knowledge and management** | How confident are you that you can manage to take care of your diabetes?  How confident are you that you are able to..  - find good ways to be physically active? - eat healthy?  - keep the weight you would like?  - measure your blood sugar as often as you need? - make changes to your medicine or treatment if you are ill?  - tell your HCP if you are having problems with your diabetes?  - use and adjust your insulin pump yourself so it suits your needs? - reach to blood sugar measurements you are your sensor or flash based measurement device  How confident are you that you..  - know how to react if your blood sugar is too high or too low? - know the right technique for injecting your medicine? - know how to dose your insulin in relation to physical activity or sports? - know how to dose your insulin in relation to meals? - have the knowledge about diabetes that you need? |
| **13. Wish for support or encouragement for self-care** | In which areas would you like particular encouragement or support:  (1. *changing my eating habits,* 2. *changing how much physically active I am, 3. taking my medicine in a way that works better for me, 4. measure my blood sugar in a way that works better for me, 5. handle my psychological or social issues, 6. changing certain habits, e.g. related to alcohol or smoking, 7. Other, 8. Not relevant at this time)* |
| **14. Symptom screening questions** | Questions about symptoms PWD may experience which may or may not be related to diabetes.  Have you been “very bothered” by the following during the past 4 weeks?  - Pain in feet or legs? - Recurring gastro-intestinal problems? - Chest pain, palpitations or shortness of breath? - Lack of enjoyment of sexual activity or difficulties completing intercourse? - Difficulties sleeping? |
| **15. Pain symptom distress** | How bothered have you been by these symptoms the past 4 weeks?  - pain in legs or feet when you walk? - pain in legs or feet at night? - pain which radiates through your legs and feet? - pain in legs or feet during the day? |
| **16. Gastro- intestinal symptom distress** | How bothered have you been by these symptoms the past 4 weeks?  - heart burn?  - constipation?  - nausea or vomiting?  - diarrhea?  - stomach pain? |
| **17. Cardiovascular symptom distress** | To what extent have you been bothered by the following symptoms the past 4 weeks? - shortness of breath in physical activities,  - difficulty breathing at night,  - palpitations or chest pain |
| **18. Sexual symptom distress** | To what extent have you been bothered by the following symptoms the past 4 weeks?  - Difficulty completing sexual intercourse?  - Difficulty getting erection? (male)  - Dryness in the vagina? (female) |
| **19. Sleep difficulty** | - How would you rate the quality of your sleep? How often have you experienced the following the past 4 weeks: - Slept badly or uneasy? - difficulty falling asleep? - waking up too early? - waking up multiple times and having hard time falling asleep again? |
| **20. Concentration, fatigue and tiredness** | To what extent have you been bothered by the following symptoms the past 4 weeks?  - concentration difficulties,  - general fatigue,  - increasing tiredness during the day. |
| **21. Foot ulcer/foot problem** | Have you had problems with foot ulcers, ingrown nails the past 4 weeks? |
| **22. Annual check-up: Eyes, Feet** | Within the past year, did you - have your feet checked by a foot therapist?  - have your eyes examined for diabetes related changes? |
| **23. Confidence in contact with HCP.** | How confident are you that you will be able to get hold of a health professional if you need assistance with your diabetes? |
| **24. Medicine satisfaction and experience** | Questions regarding medicine you take for your diabetes (e.g., insulin and other medicines to regular blood sugar, hypertension, cholesterol)  - How effective would you say your medicine is? - How much of a hassle is it to take your medicine? - Are you bothered by side effects from your medicine?  - How satisfied are you all in all with your medicine? |
| **25. Blood sugar regulation** | - How would you say your blood sugar regulation has been the past month?  How often have you  - been bothered by your blood sugar fluctuating between being too high and too low?  - been worried about getting low blood sugar? - been worried that your blood sugar is too high? |
| **26. Blood sugar unawareness** | How often do you feel symptoms of low blood sugar? |
| **27. Low BG events requiring assistance** | Number of incidents with low BG where assistance was required. |
| **28. Desired discussion topics for next visit** | Which are the most important topics you would like to talk about: Blood tests, supplies, self-care (exercise/diet/BS), medicine, diabetes complications, expectations to care, social support, education, psychological, economic challenges, goals, other: Free text. |

Table A13. Content outline of the Danish PRO diabetes questionnaire. Content overview for v1.0 of the Danish PRO diabetes questionnaire used in M-PRODIA. Items are shown for illustration and are not for sure. They are not depicted in full. The translation to English is preliminary. The corresponding author can be contacted for inquiries regarding use of the complete questionnaire for research purposes.

This is a Multimedia Appendix to a full manuscript published in the JMIR Research Protocols. For full copyright and citation information see <http://dx.doi.org/10.2196/jmir.28391>.

Developed by Aalborg University Hospital, Denmark, 2019.
