# Supplementary material for: Perceived Benefits, Barriers, and Facilitators of a Digital Patient-Reported Outcomes Tool for Routine Diabetes Care: Protocol for a National, Multicenter, Mixed Methods Implementation Study
Source: JMIR Res Protoc. 2021 Sep 3;10(9):e28391. doi: 10.2196/28391 (PMC8449301; doi:10.2196/28391)
Supplement: Multimedia Appendix 6 [file resprot_v10i9e28391_app6.docx]

**Multimedia appendix 6:
PRO-EVAL-P–Patient Evaluation of the PRO Questionnaire**

**1. How relevant were the [PRO] questions in relation to your diabetes care?**

Not at all–Very relevant (1–5). Free text.

**2. How difficulty/easy was it for you to fill out the questionnaire?**Very easy–Very difficult (1–5). Free text.

**3.** **Were there questions which were difficult to understand?**
No; Yes, but only one or very few; Yes, several. Free text.
If yes; Please describe [interactive access to questions for identification)

**4. Did you miss questions about important topics related to your life with diabetes or your diabetes treatment?**No, Yes, to some degree; Yes, to a large degree. Free text (indicate what is missing)

**5. Do you feel the questionnaire has made you more/less worried about your diabetes or your diabetes treatment?**More worried–Less worried (1–5). Free text.

**6. Were there questions in the questionnaire which you did not like to have to answer?**No; Yes, but only one or very few; Yes, several. Free text.
If yes; Please describe [interactive access to questions for identification)

**If you have other comments, praise or criticism of the content or the wording of the questionnaire kindly write them here.**

Free text

Thank you

Your input is important for the ongoing work to improve the questionnaire.

This is a Multimedia Appendix to a full manuscript published in the JMIR Research Protocols. For full copyright and citation information see http://dx.doi.org/10.2196/jmir.28391.

Developed by Aalborg University Hospital, Denmark, 2019.
