# Supplementary material for: Perceived Benefits, Barriers, and Facilitators of a Digital Patient-Reported Outcomes Tool for Routine Diabetes Care: Protocol for a National, Multicenter, Mixed Methods Implementation Study
Source: JMIR Res Protoc. 2021 Sep 3;10(9):e28391. doi: 10.2196/28391 (PMC8449301; doi:10.2196/28391)
Supplement: Multimedia Appendix 7 [file resprot_v10i9e28391_app7.docx]

**Multimedia appendix 7:**Patient Evaluation of use of PRO after a diabetes visit: PRO-CON-EVAL-P-*Short Form*.

The following questions are about your experiences of the conversation you have just had about your diabetes. Your answers are confidential and will NOT be shared with your health care professionals. It is important you answer the questions as honestly as possible

**1. Did you discuss the topics that were most important to you?**

Not at all–great extent (1–5)
Free text

**2. Did you get the treatment, advice, or help with your diabetes that you needed?**

Not at all–Great extent (1–5)
Free text

**3. Overall, how would you rate the quality of this conversation / consultation?**

Very poor–Very good (1–5)
Free text

The next questions are about your experience with the use of your answers to the diabetes questionnaire during the conversation.

**4. To what extent did your HCP use your answers to the diabetes questionnaire in your conversation?**

Not at all–Great extent (1–5)
Free text

**5. Do you feel the display of the results of your questionnaire responses gave a good picture of your current situation, needs and priorities related to diabetes?**

Not at all–great extent (1–5)
Free text

**6. Did the display of the results of your questionnaire responses help you to talk about what was most important to you?**

Not at all–Great extent (1-5)

Free text

**7. Did you experience any problems or unpleasant experiences of any kind in relation to the way your questionnaire responses were used during the conversation?**

Not at all–Great extent (1–5)
Free text

**8. Did you feel worse or better prepared for the conversation because you had answered the diabetes questionnaire?**

Worse–better (1–5)
Free text

**9. Do you feel the use of your questionnaire responses resulted in you getting to speak less or more during the conversation?**

Less–More (1–5)
Free text

**10. Do you experience that the use of the diabetes questionnaire helped you find ways to improve the way you self-manage your diabetes?**

Not at all–Great extent (1–5)
Free text

**11. Do you experience that the use of your questionnaire answers helped your health professional to find better ways to improve your diabetes treatment?**

Not at all–Great extent (1–5)
Free text

**12. How interested would you be in using the diabetes questionnaire again at future diabetes visits if it becomes possible?**

Not at all–Great extent (1–5)
Free text

**13. If you have other comments, praise, or criticism regarding the diabetes questionnaire kindly write them here.**

Free text

Thank you for completing this questionnaire!

This is a Multimedia Appendix to a full manuscript published in the JMIR Research Protocols. For full copyright and citation information see <http://dx.doi.org/10.2196/jmir.28391>.

Developed by Aalborg University Hospital, Denmark, 2019.
