# Supplementary material for: Perceived Benefits, Barriers, and Facilitators of a Digital Patient-Reported Outcomes Tool for Routine Diabetes Care: Protocol for a National, Multicenter, Mixed Methods Implementation Study
Source: JMIR Res Protoc. 2021 Sep 3;10(9):e28391. doi: 10.2196/28391 (PMC8449301; doi:10.2196/28391)
Supplement: Multimedia Appendix 8 [file resprot_v10i9e28391_app8.docx]

**Multimedia appendix 8:**Semi-structured interview guide for interviews with PWD (topic outline)

**Introduction (excerpt)**

- This interview is part of a national research evaluation study about the PRO Diabetes Questionnaire. It will be recorded for research purposes. All results are only used anonymously.
- Our purpose is to hear from you regarding how it was to fill out the questionnaire and how you experienced the use of your questionnaire responses affected your scheduled consultation/conversation.
- This interview is not an exam, and I am not judging you, the healthcare professionals, and any persons you mention during the interview will be anonymized.
- Therefore, you should not hold back on mentioning things you are in doubt about if things were hard to understand.
- Also, even if you think others might have understood it or done it in a different way than you.

**Personal background**

Information is obtained regarding type of diabetes, medicine, complications, comorbidities, education, living status.

***Context and Setting***

- Context for getting the questionnaire
- Where was it filled out? Why? What were expectations?
- How did it go? Did anyone assist?
- General experiences with use of IT for health compared to this experience

***Experience with Completing the Questionnaire***

- Difficulty, understanding, relevance,
- Rating of the quality of questionnaire
- Experience of completing the questionnaire—negative, neutral, positive?
- Impact and effects of filling out the questionnaire?

***Preparation for Diabetes Visit***

- How do you usually prepare for a diabetes visit?
- Did you prepare differently as a result of completing the questionnaire?
- Any changes in your expectations or attitudes regarding your visit?

***During the Diabetes Visit***

- What was important for you to achieve at this visit?
- Had the HCP seen your PRO answers before your visit?
- How did the HCP use your PRO results?
- How did the HCP share your PRO results with you?
- How did the PRO screen reflect your current diabetes situation, your issues, and your priorities?
- What was important for you to cover in this visit? Did you cover all important matters?
- Did your HCP propose treatment or care related to the things that were identified?
- Overall, how was the conversation with your HCP, and how do you feel PRO influenced this?

***Specific Issues***

- Specific topics/research questions—Topics identified in accordance with the formative implementation research and evaluation process.
- For examples: How do you feel about the inclusion of questions about sexual problems in the questionnaire? How did you feel about the display of this item on the screen in the visit?

***Conclusions***

- Overall, how would you describe your combined experience of completing the diabetes questionnaire and its use in your diabetes visit?
- What was the most positive aspects for you?
- Did you experience anything negative or bad related to the use of the questionnaire?
- How interested would you be in using it in the future—in different settings—and why?
- Suggestions and advise for the project in the future? Areas for improvement?
- Anything else that is important to share regarding the questionnaire or its use?

This is a Multimedia Appendix to a full manuscript published in the JMIR Research Protocols. For full copyright and citation information see <http://dx.doi.org/10.2196/jmir.28391>.

Developed by Aalborg University Hospital, Denmark, 2019.
