# Supplementary material for: Perceived Benefits, Barriers, and Facilitators of a Digital Patient-Reported Outcomes Tool for Routine Diabetes Care: Protocol for a National, Multicenter, Mixed Methods Implementation Study
Source: JMIR Res Protoc. 2021 Sep 3;10(9):e28391. doi: 10.2196/28391 (PMC8449301; doi:10.2196/28391)
Supplement: Multimedia Appendix 9 [file resprot_v10i9e28391_app9.docx]

**Multimedia appendix 9:**Main background and sociodemographic variables for PWD.

These data are collected using different methods according to feasibility at each study site.

1. Age
2. Gender
3. Municipality
4. Type of diabetes
5. Duration of diabetes
6. Highest education
7. Living status (with others/living alone)
8. Occupation/employment type (employed, student, self-reliant, retired, on unemployment benefits, other)
9. Diabetes complications (none, retinopathy, neuropathy, nephropathy, foot problems, sexual dysfunction, cardiovascular disease)
10. Chronic illness comorbidity

This is a Multimedia Appendix to a full manuscript published in the JMIR Research Protocols. For full copyright and citation information see <http://dx.doi.org/10.2196/jmir.28391>.

Developed by Aalborg University Hospital, Denmark, 2019.
