# Supplementary material for: Perceived Benefits, Barriers, and Facilitators of a Digital Patient-Reported Outcomes Tool for Routine Diabetes Care: Protocol for a National, Multicenter, Mixed Methods Implementation Study
Source: JMIR Res Protoc. 2021 Sep 3;10(9):e28391. doi: 10.2196/28391 (PMC8449301; doi:10.2196/28391)
Supplement: Multimedia Appendix 10 [file resprot_v10i9e28391_app10.docx]

**Multimedia appendix 10:**HCP Baseline Profile Questionnaire.

*Completed by all HCP at beginning of the study.*

1. **What is your age group?**18–40
   41–60
   ≥ 61
2. **What professional group do you belong to?**

Physician (specialist)
Physician (not specialist)
Nurse
Dietitian
Psychologist
Physiotherapist
Social and health counsellor
Other:

**3. Are you specialized in diabetes?**Yes/No

**4.** **How many patients do you typically see per week?**
1–4
5–10
>10

**5.** **How many years have you worked in the healthcare system?**

1–5, 5–10, 10–15, 15+?

**6. How many years of experience do you have with providing care for PWD?**

1–5, 5–10, 10–15, 15+?

**7. What level of experience do you have using the patient’s answers to a PRO questionnaire in a consultation?**

- None
- Sporadic
- Some experience
- Quite a bit of experience
- A lot of experience

**12. Have you been involved in the development of the PRO diabetes questionnaire or the design of the PRO IT solution?**

Yes/No

**13. Have you completed special training (courses, training, supervision) in use of patient-centered communication techniques in patient consultations during the past 5 years?**

Yes/No

**14. What training did you receive regarding how to use the patient’s questionnaire responses in your dialogue with your patients?** (*You can mark more than one option*.)

I did not get any training
I have received informal support from colleagues
I have taken part in training/workshop for less than 1 hour
I have taken part in training/workshop for up to 2–3 hours
I have taken part in training/workshop for at least 4 hours
Other: Free text

**15. How confident do you feel at this time point that you can use the patient’s PRO answers**

**(as shown on your PRO dashboard) in a good way in your dialogue with your patients?**Not at all confident–Very confident (1–5)

This is a Multimedia Appendix to a full manuscript published in the JMIR Research Protocols. For full copyright and citation information see <http://dx.doi.org/10.2196/jmir.28391>.

Developed by Aalborg University Hospital, Denmark, 2019.
