# Supplementary material for: Perceived Benefits, Barriers, and Facilitators of a Digital Patient-Reported Outcomes Tool for Routine Diabetes Care: Protocol for a National, Multicenter, Mixed Methods Implementation Study
Source: JMIR Res Protoc. 2021 Sep 3;10(9):e28391. doi: 10.2196/28391 (PMC8449301; doi:10.2196/28391)
Supplement: Multimedia Appendix 11 [file resprot_v10i9e28391_app11.docx]

**Multimedia appendix 11:
HCP evaluation of use of PRO: PRO-CON-EVAL-HCP including algorithm review form.**

*Completed by the HCP after use of PRO in a diabetes visit.*

These questions are about your experience of the conversation, and how the diabetes questionnaire was used and affected the conversation. The questions are answered on a scale from 1to 5. Notice which direction the scale has. Please explain your answers briefly in the free text field underneath each question to the extent it is possible.

**Type of diabetes visit** (if applicable).

**Attendance of caregiver**: Yes/No.

**1. To what degree did you use the display of the patient’s PRO results in the conversation?**

Not at all–very large degree (1–5)

Elaboration:

**2. Did you get to talk about all the topics that you feel were important for this patient?**

Not at all–large degree (1–5)

Elaboration:

**3. What effect would you say the use of patient’s PRO answers had on the quality of the conversation?**Very negative–very positive (1–5)
Elaboration:

**4. Did you experience problems or challenges in the conversation due to the use of the patient’s PRO answers?**

Not at all–large degree (1–5)

Elaboration:

**5. How useful was the display of the patient’s PRO answers on the screen during this conversation?**

Not at all useful–Very useful (1–5)

Elaboration**:**

**6. How useful was the information about options for action and dialogue for each of the patients’ PRO results during this conversation? (Diaprofil)**

Not at all useful–Very useful (1-5))

Elaboration**:**

**7. Were there any color codes from the patient’s answers which you believe are misleading or not usable?**

No, Yes →

If Yes, please complete the algorithm evaluation form to indicate which color codes and items it is.

**8. If you have other comments regarding the use of the patient’s PRO answers in this conversation, please write them here:**

Free text.

HCP Questionnaire Algorithm Review Form

*[Completed by the HCPs online when the color-codes and questions are causing problems]*

1. **What question is it about?**

[Interactive/Drop-down/paper]

**2. Problem type:**

- A. Problem with color code.

- B. Other problem

**A: If problem with color code:**

**3. Describe the problem:**

Free text

**4. What color code would be correct for this question and response option?**

Green; Yellow; Red; Do not know

**B: If other problem:**

**5. Describe the problem with using this question during your dialogue with the patient:**

Free text

This is a Multimedia Appendix to a full manuscript published in the JMIR Research Protocols. For full copyright and citation information see http://dx.doi.org/10.2196/jmir.28391.
Developed by Aalborg University Hospital, Denmark, 2019.
