# Supplementary material for: Perceived Benefits, Barriers, and Facilitators of a Digital Patient-Reported Outcomes Tool for Routine Diabetes Care: Protocol for a National, Multicenter, Mixed Methods Implementation Study
Source: JMIR Res Protoc. 2021 Sep 3;10(9):e28391. doi: 10.2196/28391 (PMC8449301; doi:10.2196/28391)
Supplement: Multimedia Appendix 12 [file resprot_v10i9e28391_app12.docx]

**Multimedia appendix 12:
Semi-structured guide for HCP evaluation. (Outline of evaluation workshop version)**
HCP evaluation workshops with HCPs were undertaken half-way and at end of the study.
Using a formative approach, end-of-study workshops with HCP were adjusted based on insights from half-way workshops and assessment of gaps in knowledge. This table provides only an outline of key questions addressed. An interview version is also available for interviews of HCP when required to supplement HCP evaluation workshops.
 **Adoption and Implementation**

How is PRO diabetes used in the practice?
How do you handle if a patient has many flagged issues?

What are the facilitators and barriers for good use (management, training, IT, other)?

What facilitates your use of PRO diabetes?
What is a barrier to your use of PRO diabetes?

**Reach**

What proportion of PWD are offered, participate and benefit from PRO diabetes?
Which PWD are not getting or benefiting from PRO diabetes ? Why?

**Effectiveness**

How does PRO create value for your patients? for the care quality ?
What are the conditions and mechanisms?
How does it work as a dialogue tool?
How does it work as a care planning tool?
How does it work as a tool for treatment decision support?

**Maintenance**

What is the potential for future use of the PRO tool at the site/for the HCP?
Arguments/rationale for continuing to use the PRO diabetes tool.
Arguments/rationale for discontinuing the use of the PRO diabetes tool.
Requirements for ensuring the PRO diabetes tool will become a future standard.

**Questionnaire and scoring algorithm validity and utility**

Appropriateness of amount and type of PRO questions?
Are there irrelevant questions, questions requiring rewording, questions missing?

For each PRO item/output: Is the wording ok? Is it working and usable in practice?
Is the scoring algorithm fitting?
Are there discrepancies between the color code output and your clinical judgment of PWD?
Are there any problems or difficulties with use of the outputs in practice?

This is a Multimedia Appendix to a full manuscript published in the JMIR Research Protocols. For full copyright and citation information see <http://dx.doi.org/10.2196/jmir.28391>.

Developed by Aalborg University Hospital, Denmark, 2019.
