# Supplementary material for: Perceived Benefits, Barriers, and Facilitators of a Digital Patient-Reported Outcomes Tool for Routine Diabetes Care: Protocol for a National, Multicenter, Mixed Methods Implementation Study
Source: JMIR Res Protoc. 2021 Sep 3;10(9):e28391. doi: 10.2196/28391 (PMC8449301; doi:10.2196/28391)
Supplement: Multimedia Appendix 13 [file resprot_v10i9e28391_app13.docx]

**Multimedia appendix 13:**
**HCP End-of-Study PRO Evaluation Questionnaire**

*Completed by all HCP at the end of the study*

All the information that you provide will be treated confidentially. Nothing that can identify you as an individual will be included in any summaries or publications of this research. Please use the open-ended response fields to add your own comments as much as possible. This information will give us the best results. Your answers are important ensure a thorough evaluation of the PRO Diabetes Questionnaire and Tool.

**1. How confident do you feel that you can use the patient’s PRO answers in a good way in the conversation today?**

Not at all confident–Very confident (1–5)

Please explain your answer: Free text.

**2. Has your way of using PRO in the conversation changed markedly during the course of the study period?**

Yes; No
Please explain your answer: Free text.

**3. Has your view about PRO diabetes changed during the course of the study period?**

I am more negative now–I am more positive now (1–5)
Please explain your answer: Free text.

**4. How does use of PRO diabetes in your diabetes visits affect your work satisfaction?**

Affects negatively–Affects positively (1–5)
Please explain your answer: Free text.

**5. Do you experience that PRO diabetes makes it more or less stressful for you to have diabetes conversations/diabetes visits?**

Less stressful–More stressful (1–5)
Please explain your answer: Free text.

**6. How interested are you in continuing to use PRO diabetes in your diabetes visits if possible?**

Not at all–Very interested (1–10)

**7. How likely is it that you will recommend PRO diabetes to colleagues who have the same type of work assignments as you?**

Totally unlikely–Extremely likely (1–10)

**8. What training/supervision have you received on how to use PRO diabetes?** (*You can mark more than one option*.).

Use “other” to use the open-ended text field if none of the available options are appropriate.

I have not participated in PRO training/workshop
I have taken part in PRO training/workshop for less than 1 hour
I have taken part in PRO training/workshop for up to 2-3 hours
I have taken part in PRO training/workshop for at least 4 hours
I have sparred with/gotten advice from colleagues with PRO experience on an ongoing basis
I have participated in general training/workshops regarding patient-centered communication
Other: Open-ended text field

**9. To what extent do you consider it is necessary that health professionals are trained in use of PRO diabetes as a dialogue tool before using it? (beyond getting basic introduction to IT solution and functionality)?**

Not necessary–Absolutely necessary (1–5)

**10. PRO diabetes can place a greater focus on psychological aspects of diabetes. Therefore, the last question is about your general attitude to this area. Please indicate below to what extent you agree with the following statement:**

*“All health professionals who provide care for or educate people with diabetes should be specially trained in identification and handling of diabetes-related psychosocial problems”*

Disagree a lot; Disagree; Neither disagree nor agree; Agree; Agree a lot

**11. If you have comments to the evaluation questions you have answered after each diabetes visit, please provide them here. We would very much like to hear them.**

Free text

Thank you for your answers!

This is a Multimedia Appendix to a full manuscript published in the JMIR Research Protocols. For full copyright and citation information see <http://dx.doi.org/10.2196/jmir.28391>.

Developed by Aalborg University Hospital, Denmark, 2019.
