# Supplementary material for: Perceived Benefits, Barriers, and Facilitators of a Digital Patient-Reported Outcomes Tool for Routine Diabetes Care: Protocol for a National, Multicenter, Mixed Methods Implementation Study
Source: JMIR Res Protoc. 2021 Sep 3;10(9):e28391. doi: 10.2196/28391 (PMC8449301; doi:10.2196/28391)
Supplement: Multimedia Appendix 14 [file resprot_v10i9e28391_app14.docx]

**Multimedia appendix 14:**
**Center Resource Profile for Person-Centered Diabetes Care**

| **Domains of the Diabetes Clinic Resource Survey Adapted for Cross-Sectorial Diabetes Care** | | |
| --- | --- | --- |
|  | **Site Resources for Support** | **Site Organization of patient Support Activities** |
| 1 | Individualized assessment of patient’s needs for self-management support and patient education | Ongoing use of data to drive quality improvement |
| 2 | Self-management support and patient education | System for documentation of self-management support and patient education |
| 3 | Development of individual treatment goals | Consistent patient input to improve quality and satisfaction with services |
| 4 | Monitoring of mental health and well-being (e.g., depression, fear, worries, stress etc.) | Integration of self-management into standard treatment |
| 5 | Coordination of psychological support/therapy related to diabetes | Multi-disciplinary team-based model for treating diabetes |
| 6 | Involvement of patients | Training of personnel within the area of self-management support and education |
| 7 | Social support | Training of personnel within the area of psychosocial issues and mental health related to diabetes. |
| 8 | Continuous care experience across primary practice, municipalities and hospital | Continuity in care |
|  | | Coordination of care and follow-up on referrals |

Outline of the baseline questionnaire completed by health care professional teams at each site at baseline. The questionnaire is adapted from the DAWN (Diabetes Attitudes Wishes and Needs) version of the Primary Care Resources For Chronic Illness Care Questionnaire (Brownson et al [56].

This is a Multimedia Appendix to a full manuscript published in the JMIR Research Protocols. For full copyright and citation information see <http://dx.doi.org/10.2196/jmir.28391>.

Developed by Aalborg University Hospital, Denmark, 2019.
